# Effects of a Comic Booklet Intervention Aimed at Preventing Second-Hand Smoke Exposure for Pregnant Women in Indonesia: A Randomised Controlled Trial

**DOI:** 10.3390/healthcare11233061

**Published:** 2023-11-28

**Authors:** Kimiko Inaoka, Ishak Halim Octawijaya, Citra Gabriella Mamahit, Jeand’arc Florentia Karundeng, Windy Mariane Virenia Wariki, Erika Ota

**Affiliations:** 1 Global Health Nursing, The School of Nursing Science, International University of Health and Welfare, Narita 286-8686, Japan; 2 Global Health Nursing, Graduate School of Nursing Science, St. Luke’s International University, Tokyo 104-0044, Japan; 22dn015@slcn.ac.jp (C.G.M.); ota@slcn.ac.jp (E.O.); 3 The School of Nutrition and Dietetics, Faculty of Health and Social Services, Kanagawa University of Human Services, Yokosuka 238-8522, Japan; 4 Global Public Health Department, Graduate School of Comprehensive Human Sciences, University of Tsukuba, Tsukuba 305-8577, Japan; 5 Kakaskasen Public Health Center, Tomohon City 95418, Indonesia; 6 Department of Community Medicine, Faculty of Medicine, Sam Ratulangi University, Manado 95115, Indonesia; wwariki@unsrat.ac.id; 7 Tokyo Foundation for Policy Research, Tokyo 106-0032, Japan

**Keywords:** behaviour change techniques, couples’ intervention, educational comic booklet, health belief model, Indonesia, pregnant women, randomised controlled trial, second-hand smoke

## Abstract

Second-hand smoke (SHS) has adverse effects for pregnant women and foetuses. This controlled and randomized clinical trial evaluated the efficacy of a comic booklet intervention in promoting SHS avoidance among pregnant women and appropriate smoking behaviours among their male partners. We allocated 140 couples to the experimental group (EG), who received the comic booklet and a reminder sticker, and 146 couples to the control group (CG), who received usual care. The primary outcomes were women’s self-reported SHS exposure and their male partners’ smoking behaviours. Secondary outcomes included knowledge and awareness of SHS. Independent *t*-tests revealed that three months post-intervention, more male partners in the EG had appropriate self-reported smoking behaviours with a small effect size (Cohen’s d = 0.35, 95% CI [0.08, 0.62], *p*-value = 0.01). Significantly more pregnant women in the EG recognised their partners’ appropriate smoking behaviours with a nearly middle effect size (Cohen’s d = 0.43, 95% CI [0.16, 0.70], *p*-value ≤ 0.01). Cues to action showed a significant difference between groups with a small effect size (Cohen’s d = 0.36, 95% CI [0.09, 0.63], *p*-value = 0.01), as evaluated by male partners. These findings suggest that the comic booklet intervention might be effective against SHS exposure by providing several cues to action through knowledge and awareness of SHS.

## 1. Introduction

Maternal exposure to second-hand smoke (SHS) during pregnancy can have detrimental consequences, including preterm birth [1], decreased placental weight [2], higher cord blood cotinine levels, and increased risk of miscarriage [3] and stillbirth [4], compared to pregnant women not exposed to SHS [2]. Furthermore, for the foetus, SHS increases the risk of congenital malformation [5], low birthweight [2,6], smaller head circumference [6], shorter length [2], and being small for gestational age [7]. Zeng and Li found that, among non-smokers, SHS exposure is related to mental health problems, especially depressive symptoms and psychological distress [8]. The developmental origin of health and disease theory [9] posits that the environment during the foetal development period influences the risk of onset for non-communicable diseases, and this claim is supported by various studies [10,11]. Furthermore, Baker [10] and Smith [11] have substantiated a link between preterm birth and low birthweight and the onset of coronary heart disease and its risk factors, including arteriosclerosis-related lesions, diabetes, and high blood pressure.

In 2020, the South-East Asian region had higher average prevalence smoking rates (approximately 48.0%) among males [12]. In 2018, the World Health Organization (WHO) estimated that 60.4% of Indonesian men smoke daily [12]. In 2017, a large-scale nationally representative survey revealed that 78% of mothers who reported SHS exposure were exposed to it at home [13]. As a public health initiative, the Indonesian Ministry of Health conducted health education advertising campaigns using shadow puppet theatres to create awareness regarding the harmful effects of both active and passive smoking on pregnant mothers and their foetuses. The Indonesian government has also disseminated infographics that highlight the dangers of SHS exposure for women and children [14,15,16].

Several systematic reviews have explored methods to reduce SHS exposure for children and pregnant women. Behbod et al. [17] reported the effectiveness of interventions in 24 of 78 studies aimed at family members and carers for reducing children’s SHS exposure. These studies applied ‘objective measure[s] of children’s SHS exposure, in-person counselling, motivational interviewing, telephone counselling, multi-component counselling-based interventions, multi-component education-based interventions, school-based strategies, educational interventions including picture books, smoking cessation, and brief intervention’ [17] (p. 2). However, these interventions were evaluated as low quality due to their high risk of bias [17]. The studies for which no statistically significant differences were found employed ‘more intensive counselling, feedback of biological measure of children’s SHS exposure, feedback of maternal cotinine, telephone smoking cessation, [and] educational home visit[s]’ [17] (p. 2).

Tong et al. [18] and Nwosu et al. [19] conducted systematic reviews on interventions to reduce SHS exposure among non-smoking pregnant women. Tong et al. [18] did not find either pharmacological or psychosocial interventions to be effective in preventing SHS exposure among pregnant women in prenatal care settings, owing to the low-quality study designs, as evaluated based on United States Preventative Task Force criteria. Nwosu et al. [19] reviewed nine individual- and household-level interventions aimed at preventing pregnant women’s SHS exposure that ‘employed educational intervention using direct teaching or counselling, brochures, posters, role-play, and/or video’ [19]. Various measurements were used to assess the interventions, including self-reported behaviours, number of cigarettes smoked, and biochemical markers of SHS exposure in pregnant women [19]. Among the nine included studies, only two were evaluated to have a low risk of bias, as most did not report information on blinding procedures, concealment of allocation, or study population selection [19].

Dherani et al. [20] reported that behaviour change techniques (BCTs) were effective in reducing SHS at home for pregnant women; however, the study methods were weak (e.g., self-reported exposure, lack of an objective outcome assessment, short follow-up period, no control group).

Satyanarayana et al. [21] conducted a systematic review and a modified Delphi survey with international experts to identify effective BCTs for preventing SHS exposure of pregnant women at home, and found that experts selected seven BCTs (e.g., measuring cotinine and providing feedback regarding the targets, spreading awareness about the health consequences of SHS, smoking limitations at home, and social and environmental effects of SHS exposure, showing the barriers to SHS prevention inside homes, and teaching problem solving).

Out of six randomised controlled trials (RCTs) [22,23,24,25,26,27] on SHS exposure prevention for pregnant women, Chi et al. [22] conducted an educational program based on the health belief model (HBM) [28,29,30] in Taiwan, using a book and follow-up telephone calls. They found that this intervention empowered pregnant women to confront smokers in their households, leading to decreased SHS exposure.

Developed in the 1950s, the HBM focuses on individual health behaviours and includes six key components that contribute to taking action to prevent or control health conditions: perceived susceptibility, perceived severity, perceived benefits, perceived barriers, cues to action, and self-efficacy [30]. Modifying factors that influence individual beliefs and health actions include age, gender, ethnicity, personality, socioeconomic status, and knowledge [30].

WHO has urged healthcare providers to offer couple-focused interventions to prevent at-home SHS exposure in pregnant women [31]. Couples who received treatment together have been shown to present better long-term adjustment for health problems: ‘There was a positive association between the quality of the relationship and the patient’s adjustment’ [32] (p. 68). Hence, a partner’s participation in an intervention is necessary for successful outcomes. Furthermore, WHO [31] reported a structured program for SHS exposure prevention during pregnancy that included educational materials developed by national organisations with jurisdiction over health and medical care. However, these materials were written in English and most of the content was not specific to SHS exposure (i.e., passive smoking) in pregnant women [33,34,35,36,37,38].

In the rheumatology field, Moll [39] found that patients who read illustrated educational books, such as cartoon- and matchstick-illustrated materials, received higher medical knowledge scores compared with patients who did not read illustrated books. In a study on acquired immunodeficiency syndrome (AIDS), comic books were reported as effective in visually engaging people, using graphics to explain educational content in a narrative and maintaining their interest [40]. Two previous studies [39,40] that used comic book interventions for other reasons reported positive outcomes, such as behaviour changes, improved knowledge, and better treatment outcomes. However, interventions using comic books in the context of smoking cessation or SHS exposure have not been examined in detail.

Comic books provide narrative experiences for readers and graphically present essential information. Using comic books for health education is becoming culturally acceptable; for example, comic books are being increasingly adopted in Indonesian society [41]. Indonesian comic book artists are often inspired by foreign styles, especially Japanese manga, which was imported to the country and translated into Indonesian beginning in the early 1990s [41]. Therefore, as Japanese comics are familiar to Indonesians, they could be used for health education to prevent at-home SHS exposure among pregnant women. However, at present, there are no educational materials utilising visual storytelling or comic book-style educational formats for preventing SHS exposure during pregnancy.

In a preliminary study to develop an educational comic booklet, Inaoka et al. [42] first searched research references and coded educational contents according to six components of the HBM: perceived severity, susceptibility, benefits, barriers, cues to action, and self-efficacy. Second, an educational comic booklet was drafted that uses strategies (peripheral, evidential, linguistic, constituent-involving, and socio-cultural strategies) to enhance cultural appropriateness [43], which was then illustrated by a Japanese manga artist in Japanese. The comic was translated into Indonesian by a bilingual Indonesian translator. Then, 17 Indonesian participants assessed the suitability of the draft-version of the educational comic booklet using a questionnaire (Suitability Assessment of Materials tool created by Doak et al. [44]) with 22 items categorized across six domains (content, cultural appropriateness, graphic illustrations/lists/tables/charts, layout and typography, learning stimulation and motivation, literacy demand). Approximately 80% of participants rated the comic as superior because it provided clear content using the graphics and because of its cultural appropriateness. Based on the results of the questionnaire, the following modifications were made to the educational comic booklet: (1) reading order; (2) one scene depicted in the comic was changed to a factual scene; (3) the wording was changed to make it more understandable. Following these modifications, the educational comic booklet was finalized.

The purpose of this study was to determine the effects of the educational comic booklet following the HBM framework, with a reminder sticker applying BCTs in reducing SHS exposure during pregnancy by increasing SHS avoidance behaviours in pregnant women and appropriate smoking behaviours in their male partners. The primary outcomes were pregnant women’s self-reported SHS exposure and their male partners’ smoking behaviours. Secondary outcomes included SHS knowledge and awareness.

## 2. Materials and Methods

### 2.1. Study Design

A two-armed longitudinal RCT was employed to assess an educational, couple-focused comic booklet intervention that applied BCTs [20,45,46,47] and the HBM [28,29,30].

### 2.2. Participants

Inclusion criteria for pregnant women were as follows: 18 years or older, non-smoking in their first trimester of pregnancy, up to 12 weeks of gestation [48,49,50,51], attending a prenatal visit at the public health centres or health posts, and exposed to SHS by their male partner (19 years or older). Inclusion criteria for male partners were smoking at least six cigarettes per week or more within two months before, or after their partner’s pregnancy [27] and living in the same household as their partner. The term ‘male partner’ was used to indicate a married or unmarried partner who met the inclusion criteria, and ‘pregnant woman’ for either relationship (married or unmarried). Exclusion criteria were pregnancy termination after the second trimester, and high-risk pregnant women with clinical illnesses, gestational diabetes, pregnancy-induced hypertension, or mental disorders [27].

### 2.3. Sample Size

The sample size was determined using G*Power 3.1.9.3 software, with a *t*-test difference between two independent means (two groups), effect size *d* set at 0.30, the critical alpha value set at 0.05 (type-I error), and a power (1-β) of 0.8 (type-II error) [52,53]. The obtained minimum sample size was 176 couples per group or 352 couples in total. Based on previous studies [22,27,54], a 15% contingency for loss to follow-up (*n* = 52) was added to the total. Therefore, the calculated number of couples in each group was 202, and the total final sample size was 404 couples. However, the sample size was ultimately smaller than the original target number, (404 couples for both groups), because the spread of COVID-19 in Indonesia since February 2020 affected the number of couples who could participate.

### 2.4. Randomisation

Research assistants identified potentially eligible pregnant women in their first trimester of pregnancy who visited public health centres and health posts for their first antenatal care (ANC) appointment. Research assistants determined participant eligibility for the study based on inclusion criteria and recruited couples in two towns in North Sulawesi, Indonesia, from March 2019 to March 2020. Research assistants informed eligible couples about the objectives, terms, common requests, inclusion criteria, exclusion criteria, and expected benefits and risks of the study. Couples received a request form and written informed consent form to become research participants at public health centres and health posts. Then, eligible couples that agreed to participate in the research were listed in a participants list. One Indonesian researcher received the names of eligible couples.

All eligible participants were assigned to either the experimental group (EG) or control group (CG) based on central randomisation. An Indonesian researcher from the research team conducted the simple random assignment using a computer-based random number generator. The Indonesian researcher provided the research assistants with the names of the couples who will receive the intervention. The principal investigator was the outcome assessor, and the Indonesian researcher who conducted the randomisation was not involved in the data analysis.

### 2.5. Interventions

At baseline, pregnant women and their partners in the EG received an educational comic booklet and a sticker as a reminder from a research assistant. The printed educational comic booklet consisted of four full-colour pages. The educational content was uniformly illustrated (offer/direct towards appropriate written materials—a BCT strategy). The comic was written in Indonesian and contained standardised information based on the following HBM components: perceived susceptibility, perceived severity, perceived benefits, perceived barriers, cues to action, and self-efficacy [42]. The unique strength of this educational comic is its application of both BCTs and the HBM. The main characters in the comic are a midwife, a pregnant woman, and her male partner. When the couple visits the prenatal care clinic, the midwife educates them on what SHS is and how to prevent it in their homes.

The comic book includes eight sections utilising BCTs and components of the HBM: (1) explanation of SHS, (2) prevalence of SHS for pregnant women in Tomohon, (3) effects of hazardous substances on pregnant women and foetuses (provide information on the consequences of SHS—a BCT strategy), (4) health risks for pregnant women and foetuses (perceived susceptibility component of the HBM), (5) characteristics of tobacco smoke, (6) benefits of preventing SHS (benefit component of the HBM), (7) barriers to SHS prevention (perceived barriers component of the HBM), and (8) countermeasures for the barriers (HBM component and a BCT strategy), and actions to prevent SHS in the home (facilitate action, plan development, and facilitate goal setting—BCTs).

Moreover, along with the educational comic booklet, a reminder sticker (cue to action—an HBM component and a BCT strategy) was used to indicate a smoke-free home. The sticker stated that smoking is not allowed inside the house, which was illustrated through a large cross with a picture of a cigarette inside the house.

The participants in the CG only received usual care from health staff at their prenatal care visit, which included regular brief advice (How to avoid smoke and how to distance themselves from smoke) that was provided to both the EG and CG. At baseline, pregnant women’s SHS avoidance and their male partners’ smoking behaviours at home were assessed through partner evaluation and self-report. Three months post-intervention, data on the same variables were collected again, on-site.

### 2.6. Study Tools

#### 2.6.1. Primary Outcomes

The primary outcomes were SHS avoidance in pregnant women and appropriateness of their male partners’ smoking behaviours at home. For pregnant women, primary outcomes were assessed using self- and partner-report questionnaires that evaluated their behavioural responses when around their smoking partners. The questionnaire included (a) the Martinelli Scale from Avoidance of Environmental Tobacco Smoke [55], as evaluated by pregnant women, and (b) a male partner’s report on their pregnant partner’s behaviours regarding SHS exposure.

The Martinelli scale asks about the extent to which SHS could be avoided in certain situations and includes items such as permitting smoking in the wife’s home and car, staying around someone who lights up, associating with smokers, and remaining in a smoking section of a restaurant. The respondents indicated their level of agreement with each statement on a four-point Likert scale ranging from 4 = almost never true to 1 = almost always true. An average of the responses for each item produced a composite score to be used in the analysis, creating an index ranging from one to four (total score from 19 to 76), with higher values indicating more avoidance of SHS exposure. The alpha reliability ranged from 0.90 to 0.93, and the stability coefficient was 0.93. Martinelli developed construct validity by comparing the scores of smokers to non-smokers. The questionnaire was validated in a sample of 95 mothers (mean age = 36) and yielded an internal consistency of 0.81 [55].

For male partners, the primary outcomes were smoking-related behavioural responses when around their pregnant partner. The questionnaire included (a) a self-report of smoking behaviours at home and (b) pregnant partner’s report on their partner’s smoking behaviours.

Except for the Martinelli Scale, which was validated by Martinelli [55], the questionnaires were initially prepared in English by the research team, based on the content of the educational comic booklet, and then translated into Indonesian. Although the questionnaires were not validated, they were independently back-translated into English to check the quality of translation before being implemented in the study.

The questionnaires developed for this study contained 27 items for pregnant women and 11 for their male partners. The respondents scored their level of agreement with each statement on a four-point Likert scale ranging from 1 to 4, with higher values indicating higher SHS avoidance by pregnant women and more appropriate smoking behaviours (preventing pregnant women’s SHS expose) of male partners, except for some items that only pertained to pregnant women (items A2, A4, A8, A9, A11, A16, A19).

#### 2.6.2. Secondary Outcomes

The secondary outcomes were SHS knowledge, health beliefs based on the HBM, and self-efficacy, assessed through self-report questionnaires for the couples. There were 38 items in the questionnaire for pregnant women and 40 in the partners’ questionnaire. Regarding SHS knowledge, respondents were asked to select either ‘yes’ or ‘no’ for each question. Correct responses received 1 point, while incorrect responses received 0 points. For health beliefs and self-efficacy, the respondents rated their level of agreement with each statement on a four-point Likert scale ranging from 1 to 4, with higher values indicating more appropriate health beliefs and higher self-efficacy. However, for perceived barriers, lower values indicated more appropriate health beliefs.

The General Self-Efficacy Scale (GSES) [56] was validated in a sample of East German migrants in 1989 and 1991 [57]. The reliability of the GSES was tested twice within a two-year period, and alphas ranged from 0.82 to 0.93 among German participants in 1989 [57] (p. 35). The retest reliability was 0.47 for men and 0.63 for women in 1991 [57] (p. 36). Concurrent validity and predictive validity were assessed for the GSES [57] (p. 36).

### 2.7. Statistical Analysis

The comic booklet intervention was the main independent variable. Demographic variables were also treated as independent variables, which were listed as background characteristics (Table 1). Confounding factors were initially examined using descriptive statistics, such as means, standard deviations, and percentages.

The dependent variables were avoidance of SHS behaviours among pregnant women and their male partners’ smoking behaviours as primary outcomes and secondary outcomes (health beliefs, knowledge, and self-efficacy). All statistical analyses were performed using the Statistical Package for the Social Sciences (SPSS) version 29 for Windows.

First, Cronbach’s alphas (α) of each domain (primary outcomes: SHS avoidance in pregnant women and appropriateness of their male partners’ smoking behaviours at home; secondary outcomes: SHS knowledge, health beliefs) were checked to measure internal consistency of each domain. Second, Little’s test of missing completely at random (MCAR) [51] was performed for all data both at baseline and three months post-intervention. Third, independent sample *t*-tests (two-tailed) were used to check for significant differences of total scores of each domain structuring primary and secondary outcomes between the EG and CG, without checking for normality based on the assumptions of the central limit theorem [58]. Fourth, paired *t*-tests were used to assess the time effects of total scores of each domain, structuring the primary and secondary outcomes between baseline and three months post-intervention. A 95% confidence interval (95% CI) value (*p* < 0.05) was considered statistically significant. Effect sizes were estimated and evaluated using Cohen’s d. [52,59,60].

### 2.8. Ethical Approval, Research Permissions, and Clinical Trial Registration

This study was conducted with approval from the Research Ethics Committee of St. Luke’s International University, Japan (18-A078), and Sam Ratulangi University, North Sulawesi, Indonesia (7383/UN12/LL/2018). This research was conducted following the guidelines of Ethical Principles for Medical Research Involving Human Subjects [61] and Ethical Guidelines for Medical and Health Research Involving Human Subjects [62].

This research was also permitted by the Indonesian government (23 November 2018), and the municipal governments of Manado (13 March 2019) and Tomohon (27 March 2019). The study participants were provided with an explanation of the study’s purpose and methods, a consent form, and a withdrawal form. Those who agreed to participate provided written informed consent.

This study was registered as a randomised clinical trial at the UMIN Clinical Trials Registry (UMIN-CTR) with the registration number UMIN000035423 (1 February 2019).

## 3. Results

### 3.1. Participants

**Figure 1:**
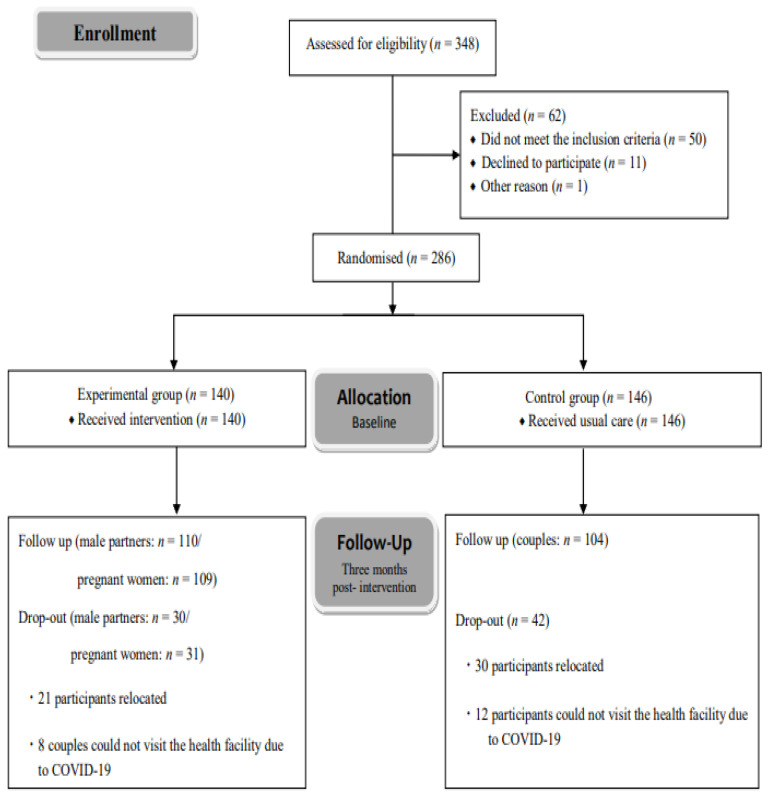
Flow diagram of the participant selection process.

Figure 1 shows the flow diagram for participant selection. For baseline analysis, of the 348 couples who consented to participate, data from 286 couples who met the inclusion criteria were analysed. The included couples were randomly assigned to either the EG (140 couples) or CG (146 couples) using a central randomisation process. Three months post-intervention, 110 male partners (79% response rate) and 109 pregnant women (78% response rate) in the EG (21–22% dropout rate), and 104 couples (71% response rate) in the CG (29% dropout rate), provided data for the primary and secondary outcomes. The final number of couples was 214 (EG: 110; CG: 104). Reasons for dropouts at three months post-intervention were that most of these participants had moved to another place, or some couples could not visit the health facility. At this point, the trial was terminated due to the COVID-19 pandemic.

### 3.2. Baseline Participant Characteristics

Table 1 shows the results for pregnant women. MCAR test’s results confirmed that the data were missing completely at random (EG: *p*-value = 0.838; CG: *p*-value = 0.247). Independent sample *t*-tests and Pearson’s chi-square tests were conducted, based on the assumptions of the central limit theorem [58], to identify significant differences in the demographic characteristics of the two groups (EG and CG).

The mean age of the pregnant women was 27.01 years (SD: 6.4) in the EG and 26.89 years (SD: 6.1) in the CG. Most pregnant women were of Minahasan ethnicity (EG: 55.0%; CG: 52.4%), had completed senior high school (EG: 62.0%; CG: 63.2%), and were Protestants (EG: 59.9%; CG: 63.2%). The mean number of gestational weeks was 15.13 (SD: 6.7) in the EG and 15.45 (SD: 6.0) in the CG. SHS at home (EG: 82.1%; CG: 77.4%) was a daily occurrence for most of the women (EG: 79.1%; CG: 71.4%). Pregnant women’s characteristics showed no between-group differences.

Regarding ethnicity and workplace, significant differences were observed in the demographic characteristics of couples who continued to participate and those who dropped out (χ^2^ = 14.93, Creamer *V =* 0.23, *p*-value = 0.02 for ethnicity; as determined using Fisher exact test), and (χ^2^ = 6.41, φ = 0.15, *p*-value = 0.04 for main workplace; as determined using Pearson’s chi-square test). Specifically, the residual analysis results showed that the dropout rate was significantly high among participants of Sangir ethnicity (adjusted residual = 2.3) and those whose main workplace was outdoors (adjusted residual = 2.2).

Table 2 shows the results for male partners. MCAR test’s results confirmed that the data were missing completely at random (EG: *p*-value = 0.574; CG: *p*-value = 0.182). Independent sample *t*-tests and Pearson’s chi-square tests were conducted, based on the assumptions of the central limit theorem [58], to identify significant differences in the demographic characteristics of both groups (EG and CG). The mean age of the male partners was 30.03 years (SD: 6.9) in the EG and 30.22 years (SD: 6.5) in the CG. Most male partners were of Minahasan ethnicity (EG: 55.7%; CG: 53.4%) and had completed senior high school (EG: 52.9%; CG: 67.8%). The mean number of cigarettes male partners smoked per day was 10.20 (SD: 6.3) in the EG, and 10.75 (SD: 7.5) in the CG. Regarding the frequency of smoking at home, most male partners smoked in their home daily (EG: 84.7%; CG: 81.0). We recruited male partners who smoked at least six cigarettes per week, as per the inclusion criteria. Male partner’s characteristics showed no between-group differences.

Regarding education, significant differences were found in the demographic characteristics of couples who continued to participate compared to those who dropped out of the study (χ2 = 12.58; Creamer V = 0.20; *p*-value = 0.01), as determined using Fisher’s exact test. The residual analysis results showed that the number of male partners who graduated from university or college and continued to participate was significantly higher compared to those who dropped out (adjusted residual = 2.8).

### 3.3. Cronbach’s Alpha Coefficients for Each Domain

Table 3 shows Cronbach’s α for each domain. For pregnant women, we found two items (D3 and H5) that should be deleted in two domains (perceived SHS-related disease susceptibility D and cue to action for preventing SHS exposure H). The α of the domain “perceived SHS related disease susceptibility D” at baseline (α = 0.73 → 0.83), and at three months post-intervention (α = 0.58 → 0.83) were improved. The α of the domain “cue to action for preventing SHS exposure H” at baseline (α = 0.82 → 0.86), and three months post-intervention (α = 0.83 → 0.88) was also improved.

For male partners, we found one item that should be deleted (D3) in one domain (perceived SHS related disease susceptibility D). Cronbach’s α of the domain “perceived SHS related disease susceptibility D” at baseline (α = 0.79 → 0.87) improved three months post-intervention (α = 0.69 → 0.81).

### 3.4. Missing Completely at Random Test Results

For the data on pregnant women (total scores per domains), MCAR was not confirmed at baseline (*p*-value ≤ 0.01) or three months post-intervention (*p*-value ≤ 0.01). For the data on male partners (total scores per domains), MCAR was not confirmed at baseline (*p*-value ≤ 0.01) or three months post-intervention (*p*-value ≤ 0.01) because the significance level was less than 0.05. Therefore, multiple imputation using linear regression was adopted for all domains of couples’ data at baseline and three months post-intervention.

### 3.5. Primary Outcome Analyses

#### 3.5.1. SHS Avoidance in Pregnant Women

Independent sample *t*-tests were conducted based on the assumptions of the central limit theorem [58] to assess differences in pregnant women’s self- (Table 4) and partner-reported pregnant women’s behaviours (Table 4). Overall, pregnant women’s self-reported SHS avoidance (Table 4) showed no differences at baseline (Cohen’s d = −0.02, 95% CI [−0.25, 0.21], *p*-value = 0.87) and three months post-intervention (Cohen’s d = 0.13, 95% CI [−0.15, 0.40], *p*-value = 0.37). For male partners’ evaluation of their pregnant partners’ SHS avoidance (Table 4), no differences were observed between groups at baseline (Cohen’s d = −0.01, 95% CI [−0.24, 0.23], *p*-value = 0.95) and three months post-intervention with a small effect size (Cohen’s d = 0.20, 95% CI [−0.07, 0.47], *p*-value = 0.15).

Paired *t*-tests were also performed to assess time effects on pregnant women’s self- and partner-reported behaviour (Table 5). Overall, the behaviour of pregnant women and their partners in both experimental and control groups did not differ between baseline and at three months post-intervention.

#### 3.5.2. Male Partners’ Smoking Behaviours

An independent samples *t*-test was conducted based on the assumptions of the central limit theorem [58] to assess differences in male partners’ self-(Table 6) and partner-reported smoking behaviours (Table 6). For self-reported smoking behaviours at baseline (Cohen’s d = 0.19, 95% CI [−0.04, 0.43], *p*-value = 0.10), no differences were observed between groups. However, self-reported smoking behaviour three months post-intervention showed a significant difference between groups with a small effect size (Cohen’s *d* = 0.35, 95% CI [0.08, 0.62], *p*-value = 0.01). For male partners’ smoking behaviour as reported by pregnant women, no significant differences were observed between groups at baseline with a small effect size (Cohen’s d = 0.21, 95% CI [−0.02, 0.44], *p*-value = 0.08); however, a difference was observed three months post-intervention with a nearly middle effect size (Cohen’s d = 0.43, 95% CI [0.16, 0.70], *p*-value ≤ 0.01).

Additionally, we conducted paired *t*-tests to assess time effects on male partners’ self-evaluated and partner-reported smoking behaviour (Table 7). Overall, only self-evaluated smoking behaviour in the experimental group showed a significant difference between baseline and at three months post-intervention with a small effect size (Cohen’s d = −0.21, 95% CI [−0.38, −0.05], *p*-value = 0.01).

### 3.6. Secondary Outcome Analyses

#### 3.6.1. Pregnant Women’s Health Beliefs and Self-Efficacy

An independent samples *t*-test was conducted based on the assumptions of the central limit theorem [58] to assess differences in pregnant women’s health beliefs and self-efficacy (Table 8). For most domains, no significant differences were observed between groups at baseline or three months post-intervention.

Paired *t*-tests were also conducted to assess time effects on pregnant women’s health beliefs and self-efficacy (Table 9). For most domains, no significant differences were observed; however, in the experimental group, barriers with a small effect size (Cohen’s d = −0.21, 95% CI [−0.38, −0.05], *p*-value = 0.01) and cue to action with a small effect size (Cohen’s d = −0.33, 95% CI [−0.49, −0.15], *p*-value < 0.01) showed significant differences between baseline and at three months post-intervention.

A cross-tabulation was conducted on pregnant women’s self-evaluated health beliefs and self-efficacy at three months post-intervention. Almost all pregnant women (91.7~100%) in both groups selected the correct answers for all SHS knowledge questions. For perceived SHS-related disease susceptibility, almost all pregnant women in both groups (EG: 95.4%; CG: 95.1%) perceived D1, ‘breathing in a room where my partner is smoking can affect foetal development and my health’ to be a health risk. Approximately 97% of the women in both groups agreed with D2, ‘cigarette smoke from smokers in a room is harmful to me and my unborn baby’. More than half of the women in both groups (EG: 60.7%; CG: 57.0%) believed D3, ‘toxic substances were released from things (clothes, furniture) in rooms where their partner had smoked’. Almost all women in both groups agreed with E1 ‘the harmful effects of SHS exposure on pregnant women’ (EG: 97.2%; CG: 95.1%) and E2 ‘their foetuses’ (EG: 99.1%; CG: 96.1%). Most women in both groups perceived four benefits of preventing SHS exposure: F1, ‘better growth for the foetus’ (EG: 93.5%; CG: 92.1%); F2, ‘better mental health for pregnant women’ (EG: 91.6%; CG: 96.1%); F3, ‘normal gestation for pregnant women’ (EG: 90.7%; CG: 88.2%); and F4, ‘reducing neonatal infants’ risks of heart disease and diabetes’ (EG: 89.8%; CG: 93.1%).

Less than half of the women in both groups perceived two barriers to preventing SHS exposure: G2, ‘no smoking norm or policy in the house’ (EG: 42.5%; CG: 43.5%); and G3, ‘difficulty in asking the partner not to smoke inside the house’ (EG: 40.6%; CG: 34.7%). More than half of the women in both groups perceived a barrier: G4, ‘smoke-free home is a risk to routine harmonious social relations’ (EG: 56.6%; CG: 55.5%). Similarly, more than half of the women in both groups agreed with four cues to action: H1, ‘knowing what SHS is’ (EG: 66.7%; CG: 58.9%); H2, ‘knowing risks of SHS for the mother’ (EG: 73.2%; CG: 59.8%); H3, ‘knowing risks of SHS for the foetus’ (EG: 77.8%; CG: 61.8%); and H4, ‘knowing how to prevent SHS exposure in the home’ (EG: 73.2%; CG: 58.8%). In the EG, almost all women (94.5%) believed that H6, ‘brief advice from research staff on preventing SHS’ was a cue to action, while 90.5% thought H7, ‘the sticker for preventing SHS’ was a cue to action.

#### 3.6.2. Male Partners’ Health Beliefs and Self-Efficacy

An independent samples-*t*-test was conducted based on the assumptions of the central limit theorem [58] to assess differences in male partners’ health beliefs and self-efficacy (Table 10). For most of the domains, no between-group differences were observed at baseline or three months post-intervention. However, at three months post-intervention, cues to action showed a significant difference between groups (Cohen’s d = 0.36, 95% CI [0.09, 0.63], *p*-value = 0.01).

Additionally, paired *t*-tests were carried out to assess time effects on male partners’ health beliefs and self-efficacy (Table 11). No significant differences were observed for most domains; however, barriers with a small effect size (Cohen’s d = −0.34, 95% CI [−0.51, −0.17], *p*-value ≤ 0.01) and cue to action with a small effect size (Cohen’s d = −0.36, 95% CI [−0.53, −0.19], *p*-value ≤ 0.01) showed significant differences between baseline and at three months post-intervention in the experimental group.

Cross-tabulations were conducted for male partners’ self-evaluated health beliefs and self-efficacy at three months post-intervention. For SHS knowledge, almost all male partners (89.3–100%) in both groups selected the correct answers post-intervention. In perceived SHS-related disease susceptibility, almost all male partners in both groups (EG: 96.4%, CG: 96.1%) perceived D1, ‘breathing in a room where I am smoking cigarettes can affect foetal development and pregnant women’s health risk’, to be true. Furthermore, 98.1% and 99.1% of the CG and EG, respectively, agreed with D2, ‘cigarette smoke from smokers in a room is harmful to pregnant women and their unborn babies’. Almost all male partners in both groups (EG: 84.4%; CG: 85.3%) agreed with D3, ‘my female partner and unborn baby breathe toxic substances that are released from things (clothes, furniture) in rooms where I smoked’. Nearly all male partners in both groups perceived E1, ‘the effects of SHS on pregnant women’ (EG: 98.2%; CG: 99.1%) and E2, ‘the foetus’ (EG: 98.2%; CG: 98%) as health risks.

Most male partners in both groups perceived four benefits of preventing SHS exposure: F1, ‘better growth for the foetus’ (EG: 88.2%; CG: 93.3%); F2, ‘better mental health for pregnant women’ (EG: 84.6%; CG: 92.3%); F3, ‘pregnant women’s normal gestation’ (EG: 83.6%; CG: 87.5%); and F4, ‘reducing neonatal infants’ risks of heart disease and diabetes’ (EG: 89.7%; CG: 93.2%). Less than half of male partners in both groups perceived four barriers to preventing SHS exposure: G1, ‘other smokers (visitors) do not accept the smoke-free home policy’ (EG: 45.0%; CG: 40.4%); G2, ‘no smoking norm or policy in the home’ (EG: 40.4%; CG: 36.5%); G3, ‘difficulty in asking other smokers not to smoke in the house’ (EG: 40.9%; CG: 47.1%); and G5, ‘losing social communication with other smokers (visitors) in the house’ (EG: 31.5%; CG: 38.3%). More than half of the male partners in both groups perceived G4, ‘a smoke-free home is a risk to routine harmonious social relations’ (EG: 53.6%; CG: 61.5%) as a barrier to preventing SHS exposure.

## 4. Discussion

Hochbaum [63] reported that ‘cues touch off behaviours when the individual is ready to behave’ (p. 8), and ‘in the external situation, [cues] such as posters, articles, and a variety of other things focus a person’s attention and feelings’ [63] (p. 8). Consistent with our results, Mayangsari and Mahmood reported that 62.5% of pregnant women (4 ex-smokers and 76 non-smokers) who were exposed to SHS had fair or good knowledge of smoking-related health risks [64]. In a qualitative study, Kaufman et al. reported that local community members had sufficient knowledge of the health risks of SHS, which they had received from tobacco control campaigns, mass media, and through health workers and family members [65], and perceived all the key components of health beliefs. Moreover, in our study, as cues to action, the educational comic booklet and sticker (reminder) enhanced well-prepared couples’ desired behavioural changes through perceived threat [29]. The sticker might help couples keep in mind the educational content learnt in the comic booklet.

Our study also showed statistical differences for certain male partners’ smoking behaviours. However, our results had a small effect size, which could have meant the intervention itself might not be as effective as we suggested. Alternatively, the weak effect sizes could have been affected by assuming that these were due to barriers (e.g., ‘spill-over’ effects). In fact, in the EG at baseline, approximately 15% of the male partners read the educational comic completely, while approximately 25.7% partially read it. In the CG, even if all participants did not receive the educational comic and sticker, some male partners reported that they ‘read the educational comic completely or partly’ at baseline and three months post-intervention. It is likely that they read other materials, such as pictures in the maternal and child health handbook, instead of the intervention comic booklet, and mistakenly answered ‘yes’ when asked if they had read it. Therefore, we were unable to confirm the actual effect size for our intervention. Other suspected factors that could have reduced the effect size are possible remaining barriers, such as risk to routine harmonious social relationships in the community [66], which over 50% of male partners in both groups mentioned. As a next step, a community-wide intervention with supportive local leaders is recommended [67].

‘An in-depth understanding of the target audience’s subjective culture is a central element in designing effective materials’ [68] (p. S125). To increase participants’ identification with the situation presented in the comic booklet, ‘skin colour and hair colour of the target group were adapted into the comic character[s]’ [42] (p. 1189). These were peripheral strategies [43] for enhancing cultural appropriateness to address our first concern that pregnant women and their male partners in the EG might not show interest in the comic booklet. Using the Indonesian language further ensured accessibility for the target audience (linguistic strategies [43]). To provide evidence (e.g., SHS rate, harmful influence on pregnant women and foetuses) to the participants as evidential strategies [43], we used eight BCTs including ‘health-related information, motivation, and behaviour skills are fundamental determinants of performance of health behaviours’ [69] (p. 84). By applying these BCTs [45], this culturally appropriate educational comic booklet might be able to provide specific action plans to avoid SHS at home (behaviour skills), extend health-related information (e.g., explanation of SHS, consequences of SHS, and risk for pregnant women and foetuses), and increase motivation (e.g., describing the benefits of SHS minus the barriers), thereby promoting behavioural changes in pregnant women and their partners.

This study has several limitations. First, owing to limits on research funding and equipment, self-report measures were used without including more objective measures, such as nicotine or cotinine levels. Chiu examined the relationship between self-reported SHS exposure and cotinine levels in the urine and blood and found that self-reported SHS exposure can provide a good estimate of biochemical markers of SHS exposure [62,70]. Thus, instead of measuring nicotine or cotinine levels, we cross-validated the self-reported measures by collecting them from both the pregnant women and their partners. Second, intention-to-treat analysis, which minimises bias when interpreting a study’s results, could not be conducted, because responses were not collected from all participants at follow-up. Dropout rates were high for both the EG (21%) and CG (28%). However, participants provided the same reasons for dropping out in both groups, which might indicate less risk of bias. Third, there are randomization approach issues (because the intervention cannot be blinded for couples as participants and evaluators). Fourth, the need analysis to employ a comic as an intervention tool did not use qualitative research methods at the same time as quantitative research. Fifth, only couples’ behavioural and attitudinal changes were confirmed as outcomes. Other outcomes such as (a) birthweight, height, gestational age at delivery, and sex (which we intended to gather as outcome measures as described in our research protocol), and (b) future disease risks (e.g., risk of respiratory disease by age 5) could not be assessed, as COVID-19 restrictions prevented couples’ access to health centres. Sixth, the sample size was smaller than the original target number (404 couples for both groups), because the spread of COVID-19 in Indonesia since February 2020 affected the number of couples who could participate.

Fifth, couples in the CG did not receive a placebo-like intervention in addition to usual care, which might have affected the follow-up rate. Seventh, in the EG, at baseline, only approximately 15% of the male partners read the educational comic booklet completely, and approximately 25.7% read it partly. Moreover, at baseline and three months post-intervention, some male partners in the CG, who did not receive the educational comic booklet and sticker, reported that they ‘read the educational comic completely or partly’. It is quite likely that they read other materials, such as pictures in the maternal and child health handbook, instead of the intervention comic booklet, and mistakenly answered ‘yes’ to the question regarding whether they had read the intervention comic booklet. Therefore, we did not analyse the changes in scores between baseline and post-intervention for both groups; only post-intervention between-group differences were analysed.

## 5. Conclusions

An HBM-based educational booklet with a sticker showed promise in being an effective intervention in SHS prevention by providing several cues to actions through hidden knowledge, and by enhancing perceptions of disease susceptibility, disease severity, benefit, and self-efficacy. The intervention was effective for male partners’ smoking behaviour (self-evaluation and peer-evaluation) (Table 6 and Table 7) and cue to action for preventing SHS exposure of male partners at 3 months post-intervention (Table 10 and Table 11). However, it was not effective for either the avoidance of environmental tobacco smoke (self-evaluation) and pregnant women’s behaviour (peer-evaluation) (Table 4 and Table 5) or for pregnant women’s health beliefs and self-efficacy at three months post-intervention (Table 8). For pregnant women in the experimental group, the time effects of barriers (Cohen’s d = −0.21, 95% CI [−0.38, −0.05], *p*-value = 0.01) and cue to action (Cohen’s d = −0.33, 95% CI [−0.49, −0.15], *p*-value < 0.01) showed significant differences between baseline and at three months post-intervention (Table 9).

To address the weak effect size, future studies should examine barriers to preventing SHS exposure, such as the risk of losing social relationships. As a next step, a community-wide intervention with supportive local leaders is recommended.

The results of this RCT can be generalised to (a) adult couples (non-smoking pregnant women and smoking male partners cohabitating) and (b) pregnant women receiving health education. Comic book interventions can be used to provide health education to target groups that use minority languages and individuals who cannot be easily educated on disease prevention with only verbal explanations. Using comic books that include essential educational content will reduce differences in content due to varying levels of knowledge among healthcare workers. Moreover, especially in perinatal care, this approach can help involve and educate partners as supporters of pregnant women. In other fields, this approach can be used for children and adults.

The authors hope that policymakers and medical personnel will use this intervention to reduce SHS exposure for pregnant women and foetuses in Indonesia. In response to COVID-19 mitigation efforts, instead of using the print version of the comic book, we suggest changing the medium of distribution to digital (e.g., video distribution), to meet social distancing requirements.

## Figures and Tables

**Table 1:** Participant characteristics at baseline (Pregnant women).

Characteristic	EG (*n* = 140)		CG (*n* = 146)		*p*-Value
	*n*	Mean (SD) or (%)	*n*	Mean (SD) or (%)	
Mean age	135	27.01 (6.4)	144	26.89 (6.1)	0.87 ^a^
Ethnicity Minahasan Sangir Mongondow Gorontalo Tinghoa Other	77 24 4 16 1 15	(55.0) (17.1) (2.9) (11.4) (0.7) (10.7)	76 21 4 21 0 21	(52.4) (14.5) (2.8) (14.5) (0.0) (14.5)	0.86 ^c^
Level of education Elementary school Junior high school Senior high school University/College	8 25 85 19	(5.8) (18.2) (62.0) (13.9)	12 28 91 13	(8.3) (19.4) (63.2) (9.0)	0.55 ^b^
Religion Protestant Catholic Muslim	82 11 11	(59.9) (8.0) (32.1)	91 8 45	(63.2) (5.6) (31.3)	0.68 ^b^
Occupation Housewife Working	108 29	(78.8) (21.2)	114 30	(78.6) (21.4)	0.95 ^b^
Workplace Indoor Outdoor Both	75 13 46	(56.0) (9.7) (34.3)	83 12 47	(58.5) (8.5) (33.1)	0.89 ^b^
Household earnings Over Rp.2,600,000/month Rp.2,600,000/month or less	58 71	(45.0) (55.0)	66 72	(47.8) (52.2)	0.64 ^b^
Type of household Nuclear family Joint family	72 64	(53.0) (47.1)	70 71	(49.3) (50.0)	0.63 ^c^
Married	125	(91.9)	133	(92.4)	0.89 ^b^
Mean number of gestational weeks	130	15.13 (6.7)	141	15.45 (6.0)	0.68 ^a^
Number of pregnancies 1 2 3, 4 or more	43 43 33 18	(31.4) (31.4) (24.1) (13.1)	38 61 30 15	(26.4) (42.4) (21.0) (10.4)	0.30 ^b^
Number of births 0 1 2 3 4 or more	11 46 36 18 12	(8.9) (37.4) (29.3) (14.6) (9.8)	10 53 40 20 11	(7.5) (39.6) (29.9) (14.9) (8.2)	0.98 ^b^
Number of children 0 1 2 3 4 or more	20 53 34 13 7	(15.7) (41.7) (26.8) (10.2) (5.5)	26 58 36 9 6	(19.3) (43.0) (26.7) (6.7) (4.4)	0.80 ^b^
Smoking status Never smoked Quit before pregnancy Quit after pregnancy	117 6 8	(89.3) (4.5) (6.1)	125 5 10	(89.3) (3.6) (7.1)	0.87 ^b^
SHS Daily Weekly Monthly Less than monthly	106 15 1 12	(79.1) (11.2) (0.7) (9.0)	100 20 1 17	(71.4) (14.3) (0.7) (12.1)	0.51 ^c^
Smoke-free home Yes No	46 88	(34.3) (65.7)	47 95	(32.9) (66.4)	0.95 ^c^

*p* < 0.05 was considered statistically significant. ^a^: *t*-test was conducted, ^b^: Pearson’s chi-square test was conducted, ^c^: Fisher exact test was conducted. EG: Experimental group, CG: Control group.

**Table 2:** Participant characteristics at baseline (Male partners).

Characteristic	EG (*n* = 140)		CG (*n* = 146)		*p*-Value
	*n*	Mean (SD) or (%)	n	Mean (SD) or (%)	
Mean age	134	30.03(6.9)	143	30.22 (6.5)	0.81 ^a^
Ethnicity Minahasan Sangir Mongondow Gorontalo Tinghoa Other	78 14 7 21 2 15	(55.7) (10.0) (5.0) (15.0) (1.4) (10.7)	78 16 3 19 0 26	(53.4) (11.0) (2.1) (13.0) (0.0) (17.8)	0.37 ^b^
Level of education Elementary school Junior high school Senior high school University/College	17 30 74 15	(12.1) (21.4) (52.9) (10.7)	14 20 99 11	(9.6) (13.7) (67.8) (7.5)	0.13 ^b^
Religion Protestant Catholic Muslim	77 13 47	(55.4) (9.4) (33.8)	89 11 44	(61.0) (7.5) (30.1)	0.84 ^b^
Occupation Private Entrepreneur Labourer Government Farmer Other	42 35 29 4 3 23	(30.0) (25.0) (20.7) (2.9) (2.1) (16.4)	49 22 29 3 8 33	(33.6) (15.1) (19.9) (2.1) (5.5) (22.6)	0.22 ^b^
Number of cigarettes smoked/day	131	10.20(6.3)	138	10.75 (7.5)	0.52 ^a^
Smoking status As usual Less after pregnancy More after pregnancy	100 30 1	(76.3) (22.9) (0.7)	109 28 3	(77.9) (20.0) (2.1)	0.64 ^b^
Smoking in home Daily Weekly Monthly Less than Monthly	116 11 0 8	(84.7) (8.0) (0.0) (5.8)	115 20 1 6	(81.0) (14.1) (0.7) (4.2)	0.18 ^b^

*p* < 0.05 was considered statistically significant. ^a^: *t*-test was conducted, ^b^: Fisher exact test was conducted. EG: Experimental group, CG: Control group.

**Table 3:** Cronbach’s alpha coefficients for each domain at baseline and three months post-intervention.

	Domains (Number of Items)	Baseline	Three Months Post-Intervention
Primary outcomes			
Pregnant women	Avoidance of environmental tobacco smoke: self-evaluation (19)	0.69	0.78
	Pregnant women’s behaviour change: peer-evaluation (3)	0.88	0.83
Male partners	Male partner’s smoking behaviour: self-evaluation (8)	0.78	0.70
	Male partner’s smoking behaviour: peer-evaluation (8)	0.69	0.70
Secondary outcomes			
Pregnant women	Knowledge of SHS C (8)	0.70	0.71
	Perceived SHS-related disease susceptibility D (2)	0.83	0.83
	Perceived SHS-related disease severity E (2)	0.94	0.93
	Perceived benefits F (4)	0.92	0.86
	Barriers to preventing SHS exposure G (4)	0.67	0.71
	Cue to action for preventing SHS exposure H (6)	0.86	0.88
	Self-efficacy I (10)	0.92	0.91
Male partners	Knowledge of SHS C (8)	0.73	0.82
	Perceived SHS-related disease susceptibility D (2)	0.87	0.81
	Perceived SHS-related disease severity E (2)	0.90	0.92
	Perceived benefits F (4)	0.91	0.87
	Barriers to preventing SHS exposure G (5)	0.72	0.68
	Cue to action for preventing SHS exposure H (8)	0.82	0.86
	Self-efficacy I (10)	0.90	0.88

**Table 4:** Comparisons of pregnant women’s avoidance of SHS exposure, as evaluated by couples at baseline and three months post-intervention.

	Experimental Group		Control Group		MD	Cohen’s d	*p*-Value	95%CI
[Baseline]	n	Mean (SD)	n	Mean (SD)				
Avoidance of environmental tobacco smoke (self-evaluation)	140	50.96 (6.29)	146	51.09 (6.09)	−0.13	−0.02	0.87 ^a^	−0.25, 0.21
Pregnant women’s behaviour (peer-evaluation)	140	9.09 (1.97)	146	9.10 (1.89)	−0.01	−0.01	0.95 ^a^	−0.24, 0.23
[At three months post-intervention]								
Avoidance of environmental tobacco smoke (self-evaluation)	109	52.17 (5.20)	103	51.38 (7.25)	0.79	0.13	0.37 ^b^	−0.15, 0.40
Pregnant women’s behaviour (peer-evaluation)	110	9.36 (1.45)	104	9.06 (1.60)	0.30	0.20	0.15 ^a^	−0.07, 0.47

Note. ^a^: *t*-test was conducted. ^b^: Welch test was conducted.

**Table 5:** Time effects of pregnant women’s avoidance of SHS exposure, as evaluated by couples.

	Baseline		Three Month Post-Intervention		MD	Cohen’s d	*p*-Value	95%CI
[Experimental Group]	n	Mean (SD)	n	Mean (SD)				
Avoidance of environmental tobacco smoke (self-evaluation)	140	50.96 (6.29)	140	52.22 (5.42)	−1.26	−0.17	0.06 ^a^	−0.34, −0.00
Pregnant women’s behaviour(peer-evaluation)	140	9.09 (1.97)	140	9.35 (1.50)	−0.26	−0.11	0.22 ^a^	−0.27, 0.06
[Control group]								
Avoidance of environmental tobacco smoke (self-evaluation)	146	51.09 (6.09)	146	51.75 (7.11)	−0.66	−0.01	0.37 ^a^	−0.26, 0.07
Pregnant women’s behaviour (peer-evaluation)	146	9.10 (1.90)	146	9.09 (1.55)	0.01	0.01	0.94 ^a^	−0.16, 0.17

Note. ^a^: Paired *t*-test was conducted.

**Table 6:** Comparisons of male partner’s smoking behaviours as evaluated by the couple at baseline and three months post-intervention.

	Experimental Group		Control Group		MD	Cohen’s d	*p*-Value	95%CI
[Baseline]	n	Mean (SD)	n	Mean (SD)				
Smoking behaviour (self-evaluation)	140	19.36 (4.49)	146	18.51 (4.19)	0.84	0.19	0.10 ^a^	−0.04, 0.43
Male partner’s smoking behaviour (peer-evaluation)	140	19.03 (3.96)	146	18.20 (3.97)	0.83	0.21	0.08 ^a^	−0.02, 0.44
[At three months post-intervention]								
Smoking behaviour (self-evaluation)	110	20.69 (4.25)	104	19.20 (4.18)	1.49	0.35	0.01 ^a^	0.08, 0.62
Male partner’s smoking behaviour (peer-evaluation)	109	20.11 (4.71)	103	18.23 (3.96)	1.88	0.43	≤0.01 ^a^	0.16, 0.70

Note. ^a^: *t*-test was conducted.

**Table 7:** Time effects of male partner’s smoking behaviours, as evaluated by couples.

	Baseline		Three Months Post-Intervention		MD	Cohen’s d	*p*-Value	95%CI
[Experimental group]	n	Mean (SD)	n	Mean (SD)				
Smoking behaviour (self-evaluation)	140	19.36 (4.49)	140	20.51 (4.35)	−1.15	−0.21	0.01 ^a^	−0.38, −0.05
Male partner’s smoking behaviour (peer-evaluation)	140	19.03 (3.96)	140	20.01 (4.86)	−0.98	−0.17	0.07 ^a^	−0.34, −0.01
[Control group]								
Smoking behaviour (self-evaluation)	146	18.51 (4.20)	146	19.44 (4.15)	−0.93	−0.19	0.05 ^a^	−0.35, −0.02
Male partner’s smoking behaviour (peer-evaluation)	146	18.20 (3.97)	146	18.51 (4.36)	−0.31	−0.01	0.56 ^a^	−0.22, 0.11

Note. ^a^: Paired *t*-test was conducted.

**Table 8:** Comparisons of each domain score on pregnant women’s health beliefs and self-efficacy at baseline and three months post-intervention.

	Experimental Group		Control Group		MD	Cohen’s d	*p*-Value	95%CI
[Baseline]	n	Mean (SD)	n	Mean (SD)				
Knowledge of SHS C	140	15.64 (0.88)	146	15.55 (1.04)	0.09	0.09	0.44 ^a^	−14, 0.32
Perceived SHS-related disease susceptibility D	140	6.60 (1.13)	146	6.57 (1.17)	0.03	0.03	0.82 ^a^	−0.20, 0.26
Perceived SHS-related disease severity E	140	6.68 (1.10)	146	6.32 (1.21)	0.36	0.31	0.01 ^a^	0.07, 0.54
Perceived benefits F	140	12.76 (2.64)	146	12.24 (2.39)	0.52	0.21	0.08 ^b^	−0.03, 0.44
Barriers to preventing SHS exposure G	140	9.51 (2.12)	146	9.86 (2.07)	−0.35	−0.17	0.16 ^a^	−0.40, 0.07
Cue to action for preventing SHS exposure H	140	15.83 (4.51)	146	15.57 (4.71)	0.27	0.06	0.63 ^a^	−0.17, 0.29
Self-efficacy I	140	31.49 (4.19)	146	30.92 (5.33)	0.57	0.12	0.32 ^a^	−0.11, 0.35
[At three months post-intervention]								
Knowledge of SHS C	109	15.72 (0.93)	103	15.84 (0.47)	−0.12	−0.16	0.25 ^b^	−0.43, 0.11
Perceived SHS-related disease susceptibility D	109	6.38 (1.05)	103	6.39 (1.01)	−0.02	−0.02	0.90 ^a^	−0.29, 0.25
Perceived SHS-related disease severity E	109	6.41 (1.02)	103	6.44 (1.06)	−0.03	−0.03	0.85 ^b^	−0.30, 0.24
Perceived benefits F	109	12.37 (1.89)	103	12.44 (1.90)	−0.06	−0.03	0.81 ^a^	−0.30, 0.24
Barriers to preventing SHS exposure G	109	10.31 (2.21)	103	10.22 (2.07)	0.09	0.04	0.76 ^a^	−0.23, 0.31
Cue to action for preventing SHS exposure H	109	17.64 (3.72)	103	16.75 (4.08)	0.90	0.23	0.10 ^a^	−0.04, 0.50
Self-efficacy I	109	31.47 (4.19)	103	31.23 (4.26)	0.24	0.06	0.69 ^a^	−0.21, 0.33

Note. ^a^: *t*-test was conducted, ^b^: Welch test was conducted. SHS = second hand smoke; C–I refers to related appendices.

**Table 9:** Time effects of each domain score on pregnant women’s health beliefs and self-efficacy.

	Baseline		Three Months Post-Intervention		MD	Cohen’s d	*p*-Value	95%CI
[Experimental Group]	n	Mean (SD)	n	Mean (SD)				
Knowledge of SHS C	140	15.64 (0.88)	140	15.73 (0.89)	−0.09	0.09	0.31 ^a^	−25, 0.08
Perceived SHS-related disease susceptibility D	140	6.60 (1.13)	140	6.40 (1.09)	0.02	0.15	0.14 ^a^	−0.01, 0.32
Perceived SHS-related disease severity E	140	6.68 (1.10)	140	6.44 (1.07)	0.24	0.17	0.07 ^a^	0.00, 0.34
Perceived benefits F	140	12.76 (2.61)	140	12.34 (1.99)	0.42	0.15	0.11 ^a^	−0.02, 0.31
Barriers to preventing SHS exposure G	140	9.51 (2.12)	140	10.18 (2.16)	−0.67	−0.21	0.01 ^a^	−0.38, −0.05
Cue to action for preventing SHS exposure H	140	15.78 (4.66)	140	17.38 (4.15)	−1.60	−0.33	≤0.01 ^a^	−0.49, −0.15
Self-efficacy I	140	31.49 (4.19)	140	31.44 (4.37)	0.05	0.01	0.93 ^a^	−0.16, −0.18
[Control group]								
Knowledge of SHS C	146	15.55 (1.04)	146	15.84 (0.62)	−0.29	−0.26	≤0.01 ^a^	−0.43, −0.10
Perceived SHS-related disease susceptibility D	146	6.57 (1.17)	146	6.43 (1.06)	0.14	0.09	0.31 ^a^	−0.07, 0.25
Perceived SHS-related disease severity E	146	6.32 (1.21)	146	6.49 (1.10)	−0.17	−0.10	0.27 ^a^	−0.27, 0.06
Perceived benefits F	146	12.23 (2.40)	146	12.45 (2.02)	−0.22	−0.07	0.38 ^a^	−0.24, 0.09
Barriers to preventing SHS exposure G	146	9.86 (2.04)	146	10.23 (2.15)	−0.37	−0.14	0.13 ^a^	−0.30, 0.03
Cue to action for preventing SHS exposure H	146	15.58 (4.69)	146	16.85 (4.30)	−1.27	−0.25	0.01 ^a^	−0.42, −0.09
Self-efficacy I	146	30.92 (5.33)	146	31.33 (4.39)	−0.41	−0.06	0.49 ^a^	−0.23, 0.10

Note. ^a^: Paired *t*-test was conducted. SHS = second hand smoke; C–I refers to related appendices.

**Table 10:** Comparisons of each domain score on male partner’s health beliefs and self-efficacy at baseline and three months post-intervention.

	Experimental Group		Control Group		MD	Cohen’s d	*p*-Value	95%CI
[Baseline]	n	Mean (SD)	n	Mean (SD)				
Knowledge of SHS C	140	17.49 (1.11)	146	17.33 (1.39)	0.16	0.12	0.29 ^a^	−0.11, 0.36
Perceived SHS related disease susceptibility D	140	6.40 (1.20)	146	6.26 (1.31)	0.14	0.11	0.35 ^a^	−0.12, 0.34
Perceived SHS-related disease severity E	140	6.31 (1.06)	146	6.34 (1.14)	−0.02	−0.02	0.86 ^a^	−0.25, 0.21
Perceived benefits F	140	12.36 (2.39)	146	12.14 (2.44)	0.22	0.09	0.44 ^a^	−0.14, 0.32
Barriers of preventing SHS exposure G	140	11.54 (2.33)	146	12.13 (2.59)	−0.59	−0.24	0.04 ^a^	−0.47, −0.01
Cue to action for preventing SHS exposure H	140	19.21 (5.61)	146	18.62 (5.14)	0.60	0.11	0.35 ^a^	−0.12, 0.34
Self-efficacy I	140	31.36 (3.80)	146	31.18 (4.89)	0.18	0.04	0.73 ^b^	−0.19, 0.27
[At three months post-intervention]								
Knowledge of SHS C	110	17.68 (0.96)	104	17.66 (1.13)	0.03	0.02	0.86 ^a^	−0.24, 0.29
Perceived SHS related disease susceptibility D	110	6.37 (0.87)	104	6.27 (0.87)	0.10	0.12	0.38 ^a^	−0.15, 0.39
Perceived SHS-related disease severity E	110	6.54 (1.02)	104	6.32 (0.88)	0.22	0.23	0.09 ^b^	−0.04, 0.50
Perceived benefits F	110	12.11 (2.43)	104	12.13 (1.71)	−0.03	−0.01	0.93 ^b^	−0.28, 0.26
Barriers of preventing SHS exposure G	110	12.74 (2.67)	104	12.88 (1.89)	−0.15	−0.06	0.64 ^b^	−0.33, 0.20
Cue to action for preventing SHS exposure H	110	21.70 (5.03)	104	19.84 (5.25)	1.86	0.36	0.01 ^a^	0.09, 0.63
Self-efficacy I	110	31.49 (3.76)	104	31.48 (3.77)	0.01	0.00	0.98 ^a^	−0.26, 0.27

Note. ^a^: *t*-test was conducted. ^b^: Welch test was conducted. SHS = second hand smoke; C–I refers to related appendices.

**Table 11:** Time effects of each domain score on male partners’ health beliefs and self-efficacy.

	Baseline		Three Months Post-Intervention		MD	Cohen’s d	*p*-Value	95%CI
[Experimental Group]	n	Mean (SD)	n	Mean (SD)				
Knowledge of SHS C	140	17.49 (1.11)	140	17.68 (1.03)	−0.19	−0.13	0.15 ^a^	−0.30, 0.04
Perceived SHS related disease susceptibility D	140	6.40 (1.20)	140	6.36 (0.87)	0.04	0.03	0.74 ^a^	−0.13, 0.20
Perceived SHS-related disease severity E	140	6.31 (1.06)	140	6.50 (1.01)	−0.19	−0.11	0.15 ^a^	−0.28, 0.06
Perceived benefits F	140	12.36 (2.39)	140	12.20 (2.43)	0.16	0.05	0.61 ^a^	−0.12, 0.21
Barriers of preventing SHS exposure G	140	11.54 (2.33)	140	12.66 (2.62)	−1.12	−0.34	≤0.01 ^a^	−0.51, −0.17
Cue to action for preventing SHS exposure H	140	19.21 (5.61)	140	21.34 (5.23)	−2.13	−0.36	≤0.01 ^a^	−0.53, −0.19
Self-efficacy I	140	31.36 (3.80)	140	31.53 (3.89)	−0.17	−0.03	0.72 ^a^	−0.20, 0.13
[Control group]								
Knowledge of SHS C	146	17.33 (1.40)	146	17.67 (1.12)	−0.34	−0.18	0.04 ^a^	−0.35, −0.02
Perceived SHS related disease susceptibility D	146	6.26 (1.31)	146	6.27 (0.88)	−0.01	0.00	0.96 ^a^	−0.17, 0.16
Perceived SHS-related disease severity E	146	6.34 (1.14)	146	6.31 (0.92)	0.03	0.02	0.84 ^a^	−0.14, 0.18
Perceived benefits F	146	12.15 (2.42)	146	12.04 (1.93)	0.11	0.03	0.70 ^a^	−0.13, 0.20
Barriers of preventing SHS exposure G	146	12.14 (2.59)	146	12.86 (2.10)	−0.72	−0.23	0.01 ^a^	−0.40, −0.07
Cue to action for preventing SHS exposure H	146	18.61 (5.14)	146	19.97 (5.04)	−1.36	−0.22	0.01 ^a^	−0.39, −0.06
Self-efficacy I	146	31.24 (4.84)	146	31.39 (3.85)	−0.15	−0.03	0.80 ^a^	−0.19, 0.14

Note. ^a^: Paired *t*-test was conducted. SHS = second hand smoke; C–I refers to related appendices.

## Data Availability

The datasets generated and/or analysed during the current study are available from the corresponding author upon reasonable request.
